# What's in a Name? The Multiple Meanings of “Chunk” and “Chunking”

**DOI:** 10.3389/fpsyg.2016.00102

**Published:** 2016-02-09

**Authors:** Fernand Gobet, Martyn Lloyd-Kelly, Peter C. R. Lane

**Affiliations:** ^1^Department of Psychological Sciences, University of LiverpoolLiverpool, UK; ^2^School of Computer Science, University of HertfordshireHatfield, UK

**Keywords:** chunk, chunking, compression, implicit learning, long-term memory, motor action, strategy, working memory

The term *chunk*, denoting a unit, and the related term *chunking*, denoting a mechanism to construct that unit, are familiar terms within psychology and cognitive science. The Oxford English Dictionary provides several definitions for “chunk.” First, “a thick, more or less cuboidal, lump, cut off anything,” or, colloquially, “a large or substantial amount.” The Merriam-Webster dictionary provides similar definitions. OUP's Oxford Dictionary alone gives a computer-related meaning: “a section of information or data.” It is in this context, a chunk as a section of information, that the word is used within psychology and cognitive science.

In these fields, a chunk typically refers to a single unit built from several smaller elements, and chunking to the process of creating a chunk. Gobet et al. (2001, p. 236) define a chunk as “a collection of elements having strong associations with one another, but weak associations with elements within other chunks.” However, in different contexts and with different authors, these two terms are used with a variety of meanings, which are very often conflated, leading to considerable confusion. Table 1 provides a taxonomy of the main meanings of “chunk” and “chunking,” which will be used to structure this article.

**Table 1:** **A taxonomy of the meanings of “chunk” and “chunking”**.

**PSYCHOLOGY**		
Memory	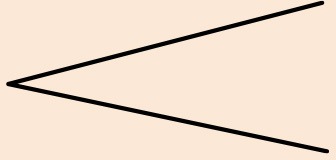	Deliberate chunking
		Automatic chunking
**PERCEPTION**		
Motoraction	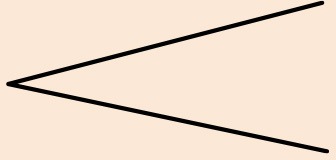	Deliberate chunking
		Automatic chunking
**COGNITIVE ARCHITECTURES**		
ACT-R		
Soar		
EPAM/CHREST		
**OTHER MEANINGS**		
Computer science		
Linguistics		
Education		

*In orange, meanings from psychology; in green, meanings related to cognitive architectures; in blue, meanings from other fields in cognitive science*.

The multiplicity of meanings of the terms “chunk” and “chunking” in the literature raises a number of questions. Do these meanings refer to the same “things”? Do they simply reflect differences in the empirical domains studied? Are the same mechanisms involved? Is learning underpinned by the same processes? As we shall see, these meanings sometimes differ in considerable ways (e.g., when referring to conscious vs. unconscious mechanisms or labeling declarative vs. procedural knowledge structures), but researchers often cite them together as if they refer to the same theoretical objects. Thus, there is the danger that, while researchers in different fields think they refer to the same theoretical concepts, they actually have different structures and mechanisms in mind. This inevitably results in lack of communication, or even worse, miscommunication.

The aim of this article is explicitly not to review the extensive literature on chunking, which would be impossible in such a short format, but to highlight some of the main meanings of the terms “chunk” and “chunking,” to discuss their commonalities and differences, and to argue that progress in our understanding of chunking will be difficult until researchers recognize these different meanings and are more precise in the way they refer to them.

## Psychology and cognitive science

### Memory

Gobet et al. (2001) distinguish between two main meanings of chunking with regard to memory: *deliberate chunking* and *automatic chunking*. Deliberate chunking is conscious, explicit, intermittent, goal-directed, and strategically intended to structure the material to memorize. This meaning is mostly used in the literature on short-term memory (also known as working memory) and is the meaning used by Miller (1956) when he provides the example of recoding binary digits in the decimal system. Conversely, automatic chunking is unconscious, implicit, and continuous. It deals with processes occurring in long-term memory and is the kind of chunking hypothesized to occur with experts when developing familiarity with a domain, for example in chess (Chase and Simon, 1973; Gobet and Simon, 1996). It is also the meaning used in several computational models, such as Competitive Chunking (Servan-Schreiber and Anderson, 1990), EPAM (Feigenbaum and Simon, 1984), and CHREST (Gobet and Lane, 2005).

Deliberate chunking can be further divided into several meanings, which often occur together. A first meaning is grouping. For example, (a a a b b b a a a) would be chunked in three groups: (a a a), (b b b), and (a a a). A second meaning, used for example by Cermak (1975), is equivalent to categorizing. For example, the list (apple car plane orange boat banana) can be chunked as (apple orange banana) and (car plane boat). A third meaning is recoding, for which Miller's discussion of recoding binary digits into decimal digits is a good example: 0000011110010011 can be recoded as 1939. A final meaning concerns using prior knowledge to reliably memorize material. For example, 1939 can be coded as “the start of World War II.”

Practically, there are important differences between the two types of chunking. Deliberate chunking leads to chunks that are fairly easy to identify, since they are explicitly defined by the chunker and can be readily illustrated or explained. By contrast, identifying chunks created by automatic chunking is more problematic, and various methods have been designed for that purpose, such as pauses in speech and eye movements in chess (for reviews, see Gilchrist, 2015; Gobet, 2015).

These two meanings are rarely distinguished in the literature; many articles start with the first meaning, and then mention the second meaning (or vice-versa), without any indication that different concepts are meant^1^. A moment of thought shows this is confusing, since three states of affairs are possible. First, both deliberate and automatic chunking are present. This is the case, for example, in many mnemonics, where the information is consciously chunked so that a long-term memory trace is created (e.g., Ericsson et al., 1980; Richman et al., 1995). Second, deliberate chunking is used in the absence of automatic chunking. For example, one might use a mnemonic to briefly memorize a phone number, without any long-term memory trace being created. Third, automatic chunking is used in the absence of deliberate chunking. This is presumably the case in many implicit-learning tasks (Berry, 1997), expertise acquisition in most fields (Gobet, 2015) and first-language acquisition (Freudenthal et al., 2007; Jones et al., 2007). For completeness sake, one can mention a final case where memory is used but neither form of chunking is involved. This would be the case, for example, when one rehearses a phone number mechanically for a few seconds without any long-term memory encoding.

The notion of compression, where a set of elements is recoded more economically (discussed by Miller in his 1956 article) is always present in both deliberate and automatic chunking. Gradients of compression exist, however. For example, with deliberate chunking, recoding 01010101 as 85 seems to use more compression than recoding (I B M) as IBM. With automatic chunking, a set of elements of arbitrary length is recoded as a single unit: rather than storing all the elements in short-term memory (STM), only a pointer is stored that denotes a chunk in long-term memory (LTM) (Newell and Simon, 1972; Guida et al., 2012).

### Perception and motor action

In the literature on perception, chunking is sometimes used with the meaning of implicit and automatic grouping of perceptual information. Thus, following the Gestalt laws of perception, objects are grouped together based on proximity, similarity, symmetry, continuity, and closure (Koffka, 1935; Gobet, 2016). Note that, in this meaning, there is no notion of memory storage.

The literature on motor action defines chunking as the learning of a complex movement sequence consisting of movement components and has studied it in a number of domains (see Rhodes et al., 2004; Diedrichsen and Kornysheva, 2015, for reviews). These include learning movement sequences (Agam et al., 2007; Cohen and Sekuler, 2010), typing (Yamaguchi and Logan, 2014), drawing the Rey–Osterrieth complex figure (Obaidellah and Cheng, 2015), drawing electricity diagrams (Lane et al., 2001), performing the discrete sequence production task (Verwey and Abrahamse, 2012; Abrahamse et al., 2013), playing the piano (van Vugt et al., 2012), speech production (Segawa et al., 2015), and sports (Shea and Wright, 2012). Typically, it is argued that motor chunks are organized hierarchically, and the production of the motor responses associated with them is unconscious and automatic. However, some research has also investigated how consciously dividing the sequence of movements to learn might lead to better skill acquisition (Fontana et al., 2009). Finally, in line with the literature on memory, Verwey and colleagues (Abrahamse et al., 2013; Verwey et al., 2015) have proposed a Dual Processor Model which distinguishes between chunks represented in an explicit format and automatic chunks.

## Cognitive architectures

The concept of a chunk is used in three leading cognitive architectures. In ACT-R (Anderson et al., 1997, 2004), a chunk is defined as unit of declarative knowledge. (Procedural knowledge is encoded as productions.) Chunks contain an “isa” field, which indicates the category to which they belong (for example numeric, textual or visual) and additional fields encoding the knowledge within the chunk (for example: “2 + 2 = 4”). Chunks have a level of activation that is a function of how recently and frequently they have been used. In Soar (Laird et al., 1987; Newell, 1990), all knowledge is encoded in procedural knowledge, and a chunk is a production (i.e., a condition–action pair). Therefore, “chunking” in this context is the mechanism by which productions are created. Thus, confusingly, a chunk is a unit of declarative knowledge in ACT-R and a unit of procedural knowledge in Soar. With CHREST (Gobet and Lane, 2005; Lloyd-Kelly et al., 2015), a chunk refers to a node in LTM. Chunking refers to the creation of such nodes, either by adding a node to the network by *discrimination*, or by adding information to an existing node, by *familiarization*. This usage follows the tradition set by the EPAM models (Feigenbaum and Simon, 1984). Thus, it can be seen that, in these three cognitive architectures, the concept of a “chunk” has totally different meanings.

## Other uses of the term “chunk”

Although, the dictionary definition refers to a computing-related meaning of chunk as a section of information or data, the term appears to be applied colloquially in computer science rather than formally. Two distinct meanings may be identified as examples: chunks as collections of information sent from one point to another, such as in distributed computing; and chunks as collections of information stored within a file.

A good example of chunks used in distributed computing is the Google File System (Ghemawat et al., 2003). Files are divided into chunks of a fixed size (64 MB) to facilitate their storage and movement between different computers (called *chunk servers*). Separation into chunks provides redundancy and makes it easier to balance the work done by tens of thousands of computers.

The PNG image format (http://www.libpng.org/pub/png/spec/1.2/PNG-Chunks.html) uses chunks to divide the information contained within a picture into sections. For example, a header chunk holds information on the width/height of the image, the number of colors, and so on; another chunk holds the color palette for the image. The image data may be a single or multiple chunks; each chunk of image data must fit in the working buffer of the image-encoding algorithm. Chunk types are also used for user-defined extensions to the PNG format, holding specialist image information, such as copyright information, text comments, etc.

In computational linguistics, chunking (also known as “light parsing” and “shallow parsing”) refers to a technique whereby a sentence is analyzed in terms of its constituents (i.e., nouns, noun groups, verbs, etc.), without specifying the internal structure and their role in the sentence. Finally, in education, chunking refers to an elementary method of division, where successive subtractions are carried out.

## Conclusions

As described in this article, the terms “chunk” and “chunking” have multiple meanings. Sometimes, the distinction is obvious, for example when memory chunks and action chunks are mentioned. At other times, the distinction is unclear, most notably when deliberate chunking and automatic chunking are mentioned with respect to memory. Finally, there are instances where mentioning diverse kinds of chunks as if they were referring to the same structures or mechanisms makes little sense, as is the case when ACT-R, Soar and deliberate chunking are mentioned together without further qualification.

The different meanings we have discussed in this article raise the question of whether different terms should be used. While polysemy (the use of the same term with different meanings) is common in everyday language and science, it is far from an ideal state of affairs. Conversely, while also common in science, synonymity (the use of different terms with the same meaning) is also problematic. Ensuring that the term “chunk” has a single meaning, at least in the context of cognitive psychology and cognitive science, would be particularly important for architects of computational chunking models who require unambiguous definitions of concepts to facilitate the development process (Gobet et al., 2015). In an ideal world, this could be achieved by constructing an ontology. Not only would this allow for precise system specifications, but it would also allow architects from diverse academic backgrounds such as computer-science, psychology and biology to communicate without ambiguity. Perhaps even more importantly, given that software development can be open-source and hence open to development by architects from different nationalities who may not share a common tongue, a precise meaning of “chunk” and other associated terms would facilitate effective system development and reduce potential friction between computational modelers.

Unfortunately, policing linguistic use is difficult, if possible at all, in science, where ideas, theories and methods are in constant flux. In fact, attempts to unify the definitions of basic concepts in psychology (e.g., De Groot, 1990) have met with little success. Thus, ambiguity may be a price to pay for the evolutionary nature of science, and this paper has limited itself to providing a taxonomy of the meanings of the terms “chunk” and “chunking.” This being said, before any understanding is met, it is important to first define the objects of research. In this respect, carefully specifying the intended meaning of terms as central as “chunk” and “chunking” is desirable for making progress in our understanding of human cognition.

## Author contributions

FG wrote the first draft, and ML and PL contributed to the following drafts.

### Conflict of interest statement

The authors declare that the research was conducted in the absence of any commercial or financial relationships that could be construed as a potential conflict of interest.

## Footnotes

^1^An additional source of confusion in the literature is that the term “chunk” is used to refer both to an element in STM and an element in LTM
